# Deciphering Antioxidant Responses in Tomato Autografts

**DOI:** 10.3390/antiox14020234

**Published:** 2025-02-18

**Authors:** Carlos Frey, Andrés Hernández-Barriuso, José Luis Acebes, Antonio Encina

**Affiliations:** 1 Área de Fisiología Vegetal, Facultad de Ciencias Biológicas y Ambientales, Universidad de León, 24007 León, Spain; cfred@unileon.es (C.F.); ahernb00@estudiantes.unileon.es (A.H.-B.); jl.acebes@unileon.es (J.L.A.); 2 Instituto de Biología Molecular, Genómica y Proteómica, Universidad de León, 24007 León, Spain; 3 Instituto de la Viña y el Vino, Universidad de León, 24009 León, Spain

**Keywords:** antioxidant capacity, catalase, lipid peroxidation, oxidative stress, redox balance, *Solanum lycopersicum*

## Abstract

Grafting is a horticultural technique that involves a healing process that requires grafted plants to develop physiological responses to overcome oxidative stress. In this study, oxidative damage, total antioxidant capacity and antioxidant enzymatic activities were analysed in functional and non-functional tomato autografts for eight days after grafting, considering scion and rootstock tissues separately. The results showed that oxidative damage, measured as lipid peroxidation, was controlled, especially in functional grafts. Scion tissues showed significant increases in total antioxidant capacity and activities of key antioxidant enzymes, including superoxide dismutase and catalase. Non-functional grafts showed elevated levels of class III peroxidase, potentially related to defensive suberisation and lignification. Principal component analysis revealed that antioxidant activities correlated dynamically with grafting stages, highlighting their critical role in stress mitigation. These results suggest that an efficient and asymmetric antioxidant response is essential for successful graft healing in tomato plants. Furthermore, different patterns in non-functional grafts underline the importance of redox balance in determining graft success.

## 1. Introduction

Grafting is a horticultural method in which two parts of a plant, typically a stem (called a scion) and the root-bearing part (called a rootstock), are cut and joined to create a single plant with combined characteristics. This technique allows desirable traits, such as disease resistance from the rootstock and superior fruit quality from the scion, to be combined [1]. Grafting is widely used in woody crops and also in herbaceous crops, such as cucurbits (melon, watermelon and cucumber) and solanaceous (pepper, eggplant and tomato) [2,3,4]. Particularly, tomato plants are grafted onto resistant rootstocks to improve their resilience under saline conditions [5,6], drought [7,8] and some diseases [9,10], among other uses.

When two stems are joined under the right conditions, the graft healing process begins, and a series of changes modulate the cut zone to form a solid graft union, a bridge between the scion and the rootstock. Previous studies have shown that graft union formation is a dynamic and continuous process involving a complex set of physiological and molecular mechanisms for successful rootstock–scion fusion [11,12,13,14,15,16]. However, despite their widespread use, some of the underlying mechanisms of graft formation remain the focus of research.

Grafting involves a stressful recovery period for the plant, and one of the challenges faced by many nurseries specialising in this production is that a significant proportion of grafted plants fail during the recovery period of healing. How plants overcome and resist this highly stressful event is complex. Cutting causes a severe injury to the stem, characterised by tissue disruption and the triggering of a wound-healing response in the close vicinity of the cutting edge. Plants have evolved mechanisms that allow them, firstly, to sense the wound-induced damage and, secondly, to activate wound protection mechanisms [17]. Much is known about the molecular mechanisms underlying wound-induced responses, including the perception of cell integrity via cell wall sensing and wound signalling via ion fluxes (Ca^2+^ or H^+^), the activation of MAPKs phosphorylation cascades, the de novo synthesis of hormones such as jasmonic acid and the production of reactive oxygen species (ROS) [17,18]. Indeed, wound response and oxidative stress-related genes are significantly correlated with grafting at very early time points (1 h after grafting, 1 HAG) [12]. Furthermore, the discontinuity created between the scion and the rootstock during grafting leads to a disruption in sap transport along the stem, drastically reducing water availability to the scion. As a result, the stomata close to optimise water use [19,20,21], leading to a decrease in CO_2_ content and detrimental effects on the photosynthetic process [20,22]. The excess photon energy beyond the photosynthetic capacity of the plant leads to the inhibition and dysfunction of photosystem II and photosystem I, disrupting the electron flow path and potentially triggering the generation of highly toxic ROS within the plant [23,24,25]. The healing conditions (high relative humidity and low light intensity) in the recovery chambers help to mitigate the effects of water stress but are not sufficient for the plant to show no change in turgor. Grafted plants are then exposed to water stress, which has been shown to induce ROS accumulation [26,27]. In addition, increased accumulation of hydrogen peroxide (H_2_O_2_) leading to oxidative damage and increased activity of some antioxidant enzymes, such as peroxidase, associated with increased accumulation of phenolic compounds have been reported in tomato incompatible plants [25].

**Figure 1:**
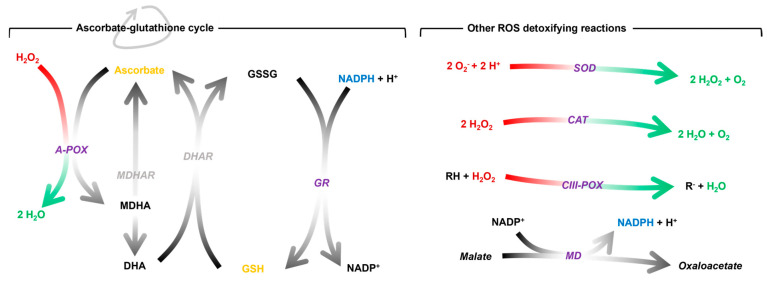
Schematic representation of the antioxidant mechanisms mentioned above and the reactions catalysed by the enzymes. The stoichiometry of the reactions catalysed by the **grey** enzymes has been omitted for simplicity. The enzymatic activities studied in this work are shown in **purple**. A-POX, ascorbate peroxidase; CAT, catalase; CIII-POX, class III peroxidase; DHA, dehydroascorbate; DHAR, dehydroascorbate reductase; GR, glutathione reductase; GSH, reduced glutathione; GSSG, oxidised glutathione; MD, malate dehydrogenase; MDHA, monodehydroascorbate; MDHAR, monodehydroascorbate reductase; NADPH, nicotinamide adenine dinucleotide phosphate; SOD, superoxide dismutase.

It is hypothesised that the stress during graft healing (mainly caused by wound syndrome and the onset of water deficit in the scion) converges in a generalised oxidative stress, particularly at the site of graft union [14,28]. To buffer this, the plant promotes an antioxidant response to prevent oxidative damage throughout the healing process. This response can be mediated by antioxidant molecules, antioxidant enzymes or a combination of both (Figure 1). The most prominent antioxidant molecules are ascorbic acid and glutathione. Both are part of the ascorbate-glutathione cycle, a biochemical process known to buffer ROS [29,30]. It is regulated by enzymes including ascorbate peroxidase (A-POX) and glutathione reductase (GR). Superoxide dismutase (SOD), catalase (CAT), class III peroxidase (CIII-POX) and malate dehydrogenase (MD) are other key enzymes in the regulation of redox homeostasis.

Considering the above, the aim of this study was to use tomato autografts to shed light on the following: (1) Whether oxidative damage occurs in the tissues adjacent to the cut area during graft healing. To this purpose, the levels of lipid peroxidation (LIPOX) in the stem tissues involved in the grafting process were monitored throughout the graft healing period (0–8 days after grafting, DAG). (2) The functional dynamics of the antioxidant machinery in the union tissues during the graft healing process. For this purpose, the total antioxidant capacity (TAC) and the activity of several antioxidant enzymes (SOD, CAT, CIII-POX, A-POX and GR) were evaluated in the same tissues as in point 1. (3) Whether there are differences in oxidative damage and antioxidant response between functional and non-functional grafts.

## 2. Materials and Methods

### 2.1. Plant Growth

Seeds of tomato (*Solanum lycopersicum* “Minibel”, Mascarell Semillas S.L., Benissoda, Spain) were imbibed for 24 h and sown in individual 200 mL containers with a moist substrate based on peat and wood fibres. The containers were covered with a plastic film to keep the humidity for four days and placed in a growth chamber at 24 ± 1 °C under light (≈40 μmol m^−2^ s^−1^) with 16/8 photoperiodic conditions and over 50–60% relative humidity. The substrate was irrigated twice a week to field capacity with Hoagland solution.

### 2.2. Grafting Technique and Healing Conditions

When the stems were over 4–5 mm in diameter (about one month old), a splice autograft with a 45° angle was made under the cotyledonary leaves: scion and rootstock were brought together, realigning vascular bundles, and tied with a graft clip (Toogoo^®^). A total of 120 grafted plants were grouped in batches of 12 plants and distributed in 10 trays (56 cm × 42 cm × 8 cm). The trays were placed under a plastic cover constituting the graft healing chamber and maintained in a growth chamber at 24 ± 1 °C. During the initial grafting phase, high relative humidity conditions were maintained (90–100%) to reduce wilting. Light conditions and relative humidity were modified during graft healing as described in [21].

### 2.3. Sampling

**Figure 2:**
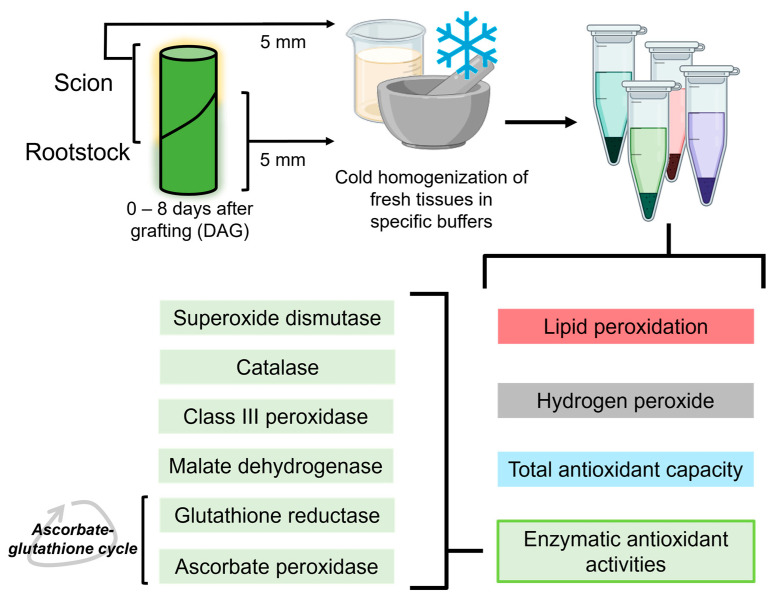
Schematic view of the experimental procedure, showing all the variables determined from the sample extracts. Created using BioRender.

On the different sampling days (0–8 DAG), five plants were randomly selected. From each plant, a 5 mm long stem segment was collected above and below the cut line (Figure 2). The 0 DAG samples corresponded to 5 mm long stem segments from non-grafted plants taken at a similar position to the grafting site on grafted plants. Non-functional grafts were identified by careful visual inspection, including the signs of lack of graft union and scion wilting.

Stem segments were homogenised at 4 °C (1 g of fresh weight/6 mL) using a cold mortar and pestle and appropriate extraction buffer. For LIPOX, TAC, SOD, CAT, MD and GR activity assays, a buffer consisting of 50 mM Tris-HCl; 0.1 mM EDTA; 0.1% Triton X-100; 2 mM DTT and 10% glycerol was used. For A-POX and CIII-POX activity assays, an extraction buffer consisting of 40 mM Tris-HCl; 1 mM EDTA and 5% glycerol was used. For the determination of H_2_O_2_, a special buffer consisting of K-phosphate buffer pH 6.4 and 5 mM KCN was used. Homogenates were incubated for 1 h at room temperature and then stored at −80 °C until use.

### 2.4. Measurement of Lipid Peroxidation

LIPOX levels were measured from malondialdehyde (MDA, a product of LIPOX) content determined by the thiobarbituric acid reaction. The extracts were centrifuged (15,000× *g*, 2 min), and 50 μL of the supernatant were transferred to a test tube, to which 50 μL of 20% trichloroacetic acid and 25 µL of 0.675% thiobarbituric acid were added. The mixture was heated to 100 °C for 20 min, rapidly cooled in a cold-water bath and centrifuged again (15,000× g, 10 min). The samples were loaded into a 96-well plate and the absorbance was measured at 600, 532 and 440 nm in a plate reader. The MDA assay required a sucrose standard line to account for the interference of soluble sugars in the assay. In this way, the concentration of sugars measured in the samples was proportional to the absorbance of sucrose at 440 nm. Thus, the concentrations of thiobarbituric acid-reactive substances) are equivalent to the concentration of MDA expressed in nmol/mL according to the following formula [31]:

$$MDA\;equivalent\left(\frac{nmol}{mL}\right)=\left(\frac{\left(A532-A600-\left(\left(A440-A600\right)\times \left(\frac{MA\;of\;sucrose\;at\;532\;nm}{MA\;of\;sucrose\;at\;440\;nm}\right)\right)\right)}{157000}\right)\times 10^{6}$$

MA: molar absorbance. MA of sucrose at 532 nm = 8.4; MA of sucrose at 440 nm = 147.

### 2.5. Measurement of H_2_O_2_ Content

To determine the concentration of H_2_O_2_ present in the samples, 30 µL of the extract was mixed with 200 µL of H_2_O_2_ reaction buffer (100 µM xylenol orange, 100 µM d-sorbitol, 250 µM FeSO_4_, 250 µM (NH_4_)_2_SO_4_ and 1% ethanol in 25 mM H_2_SO_4_). The samples were incubated in a 96-well plate for 40 min in the dark with gentle shaking. Absorbance was measured at 550 nm, and H_2_O_2_ was determined using a standard line of H_2_O_2_ (Sigma) according to the method described by Cheeseman [32].

### 2.6. Measurement of Total Antioxidant Capacity (TAC)

Total antioxidant capacity was measured using an e-BQC lab device (BioQuoChem, Oviedo, Spain), an electrochemical method based on the measurement of a redox potential [33]. The device measures the resistance of the sample to be oxidised, which is translated into antioxidant capacity. e-BQC lab distinguishes between fast (Q1) and slow (Q2) acting antioxidants, covering compounds with a higher or lower rate of free radical reduction. The instrument also calculates a QTotal value, which is the sum of both. Results were expressed in charge units (µC).

### 2.7. Measurement of Antioxidant Enzymatic Activities

The different levels of antioxidant activities in the grafted tomato stems were determined using specific methods. The frozen extracts were thawed and centrifuged (15,000× *g*, 2 min), and the supernatant was used to determine the different activities.

SOD activity was determined using the SOD assay kit-WST (Sigma, St. Louis, MO, USA), based on the measurement of the decrease in superoxide anions generated by the xanthine oxidase enzyme. CAT activity was quantified according to the method [34], based on the decrease in absorbance at 240 nm due to the decomposition of H_2_O_2_. The reaction buffer used consisted of 50 mM K-phosphate buffer pH 7 and 37.5 mM H_2_O_2_. CIII-POX was evaluated by the technique described by [35], where the absorbance of guaiacol oxidation products was measured at 470 nm. The reaction buffer used consisted of 100 mM sodium acetate pH 5.5, 0.94 mM guaiacol and 1.3 mM H_2_O_2_. MD activity was determined by the method described in [36] based on the increase in absorbance due to the conversion of NADP^+^ to NADPH in the presence of malate. The reaction buffer used consisted of 50 mM HEPES-NaOH pH 7.6, 1 mM MgCl_2_, 0.8 mM NADP^+^ and 500 mM L-malic acid. A-POX activity was quantified by the method described by [37] based on the reduction of absorbance at 290 nm due to ascorbate oxidation. The reaction buffer used consisted of 50 mM HEPES-NaOH pH 7.6, 0.2 mM ascorbate and 1.3 mM H_2_O_2_. GR activity was evaluated by the method described in [38] based on the decrease in the absorbance due to the oxidation of NADPH to NADP^+^ by conversion of oxidised glutathione to reduced glutathione. The reaction buffer used consisted of 0.1 M HEPES-NaOH pH 7.8, 4 M NaOH, 1 mM EDTA, 3 mM MgCl_2_, 0.5 mM oxidised glutathione and 0.2 mM NADPH.

### 2.8. Statistical Analyses

All data were subjected to Shapiro–Wilk and Levene tests for normal distribution and homoscedasticity. For physiological and redox parameters, all values were subjected to one-way ANOVA using IBM SPSS Statistics v. 25 software. In addition, Tukey’s test was used to compare the means of the different replicates. The statistical significance level was set at *p* ≤ 0.05. All results were expressed as mean ± standard deviation (SD). Principal component analysis (PCA) was performed with the antioxidant variables by RStudio (v. 2024.09.0+375 “Cranberry Hibiscus”) using the packages Factoextra [39], FactoMineR [40] and Gplots [41].

## 3. Results

### 3.1. Oxidative Damage Measured as Lipid Peroxidation

To monitor the effect of putative oxidative stress in grafted tissues, LIPOX was measured in both the scion and rootstock sampling tissues in the close vicinity of the graft zone throughout grafting. Tomato autografts are a highly compatible grafting model that has allowed the establishment of a time course of graft healing in tomato, with a wound response phase at 1–2 DAG, a callus formation and adhesion phase at 3–6 DAG and vascular reconnection at 8 DAG [42].

**Figure 3:**
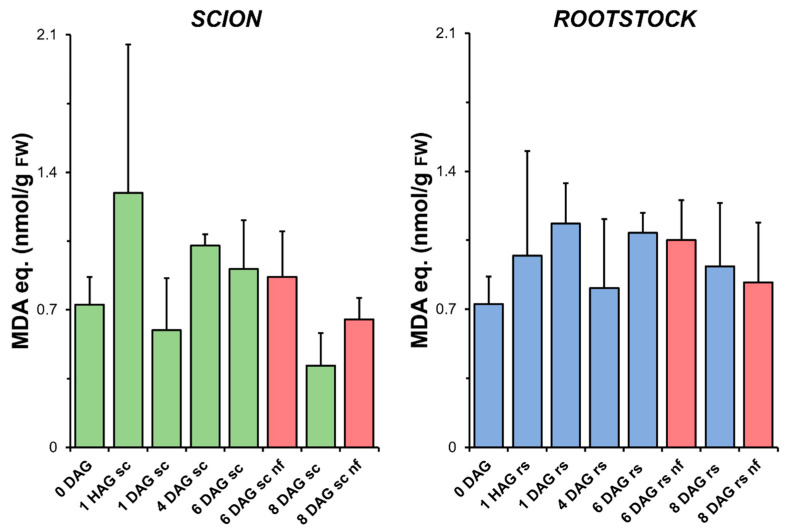
Variation in the level of lipid peroxidation measured as the production of malondialdehyde (MDA) equivalents relative to fresh weight (FW) in scion (sc, **green**) and rootstock (rs, **blue**) from 0 to 8 DAG throughout the grafting process. **Red** columns show non-functional grafts (nf). Mean ± SD (n = 5). No significant differences at the *p*-value < 0.05 level were detected after ANOVA analysis of variance followed by Tukey’s test.

As shown in Figure 3, the level of LIPOX did not change significantly during grafting and was not significantly different from the LIPOX measured in the non-grafted plants. At very early times after grafting (1 HAG), an apparent increase in the level of LIPOX of the scion tissues was observed. However, the results obtained did not allow significant differences to be established with respect to the non-grafted controls (0 DAG) due to the high variability.

In addition, no higher levels of LIPOX were found in the scion and rootstock tissues corresponding to the non-functional grafts compared to the non-grafted plants or grafted plants at the same stage of grafting. The levels of H_2_O_2_ detected in the tissues also decreased during the healing process, although the non-functional grafts had higher levels than the functional grafts (Figure S1).

### 3.2. Total Antioxidant Capacity in Grafted Tissues

**Figure 4:**
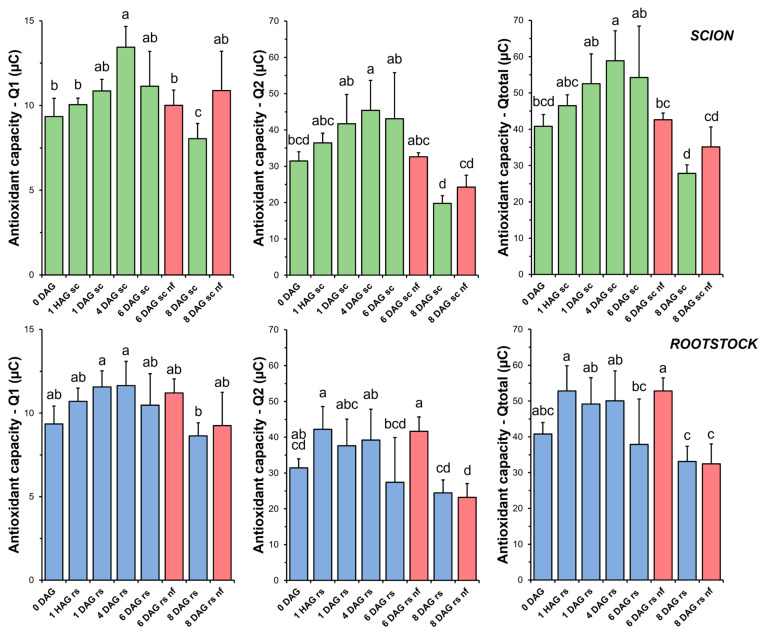
Total antioxidant capacity (TAC) in scion (sc, **green**) and rootstock (rs, **blue**) from 0 to 8 DAG throughout the grafting process. Q1 indicates the short-term TAC and Q2 the long-term TAC. Qtotal is the sum of Q1 and Q2. **Red** columns indicate non-functional grafts (nf). Mean ± SD (n = 5). Different letters indicate significant differences at the *p*-value < 0.05 level after ANOVA analysis of variance followed by Tukey’s test.

TAC in the scion tissues increased sharply soon after grafting (Figure 4). At 4 DAG, the antioxidant capacity of the scion tissues was significantly higher than that of the non-grafted plants (0 DAG). Thereafter, a decrease in TAC was measured up to 8 DAG. Both short-term (Q1) and long-term (Q2) antioxidant capacities, representing the contribution of fast- and slow-acting antioxidants, respectively, reflected the changes observed in TAC. However, our results showed that the antioxidant capacity of the slow-acting antioxidants (Q2) was greater than that of the fast-acting antioxidants (Q1).

TAC analysis on the rootstock samples showed less variation throughout the grafting process (Figure 4). Overall, no significant differences were found when comparing the non-functional grafts with the non-grafted or grafted plants at the same stage of grafting.

### 3.3. Assay of Antioxidant Enzymes Activities

In order to gain insight into the contribution of the enzymatic machinery to the antioxidant status of the grafted tissues, a time course of changes in activities of antioxidant enzymes (SOD, CAT, CIII-POX, MD, A-POX and GR) was carried out in both the scion and rootstock tissues.

### 3.4. Superoxide Dismutase

**Figure 5:**
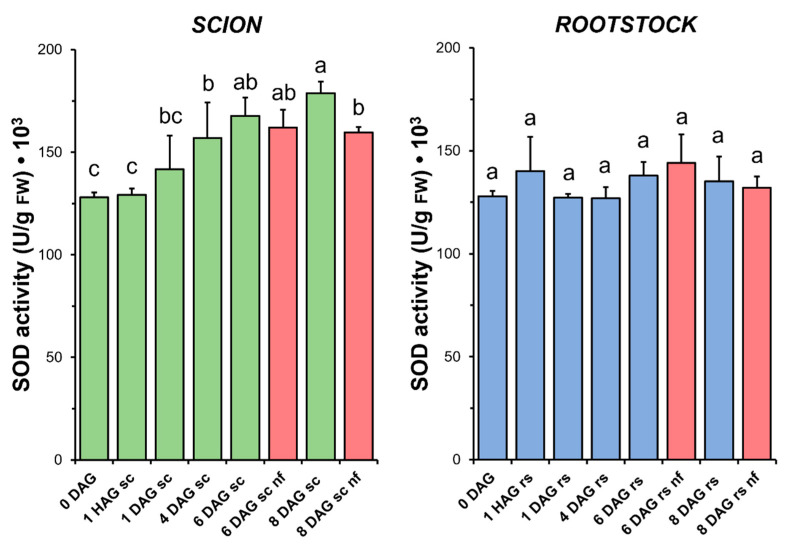
Superoxide dismutase (SOD) activity in scion (sc, **green**) and rootstock (rs, **blue**) from 0 to 8 DAG throughout the grafting process. FW, fresh weight. **Red** columns show non-functional grafts (nf). Mean ± SD (n = 5). Different letters indicate significant differences at the *p*-value < 0.05 level after ANOVA analysis of variance followed by Tukey’s test.

Concurrent with the increase in TAC observed in the scion tissues (Figure 4), a significant increase in SOD activity (Figure 5) was measured during the sampling period. Interestingly, in the scion tissue, SOD activity at 8 DAG was significantly reduced in the non-functional grafts compared to the functional grafts. In the case of the rootstock, SOD activity did not change significantly after grafting. Similarly, no significant differences were found when comparing the non-functional grafts with the non-grafted or grafted plants at the same stage of grafting.

### 3.5. Catalase

**Figure 6:**
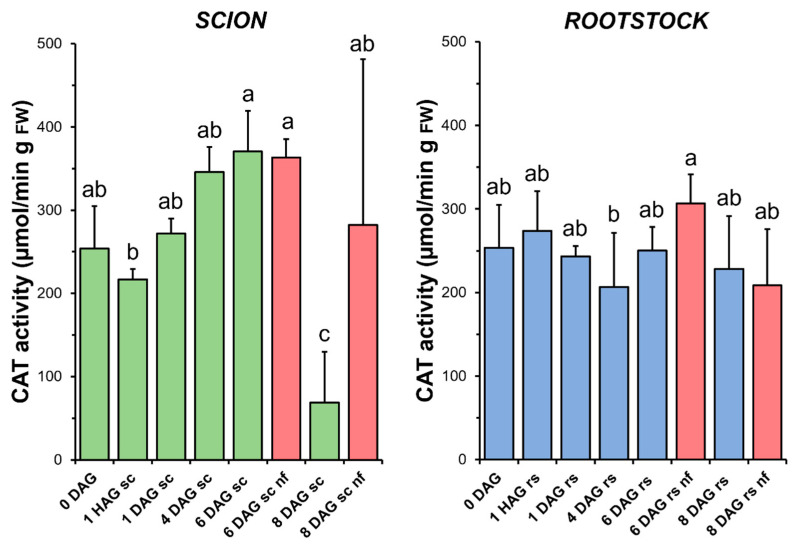
Catalase (CAT) activity in scion (sc, **green**) and rootstock (rs, **blue**) from 0 to 8 DAG throughout the grafting process. FW, fresh weight. **Red** columns show non-functional grafts (nf). Mean ± SD (n = 5). Different letters indicate significant differences at the *p*-value < 0.05 level after ANOVA analysis of variance followed by Tukey’s test.

Although an increasing trend was observed, no statistical differences in CAT activity (Figure 6) were found between the non-grafted and grafted tissues (both scion and rootstock) over the time window when a measurable increase in TAC was observed (1 HAG to 4 DAG in Figure 4). At 8 DAG, a significant decrease in CAT activity was observed in the scion tissues. Interestingly, this sharp decrease in CAT activity coincided in time with a significant decrease in TAC in the same tissues. In the rootstocks, no significant differences were found when comparing the non-functional grafts with the non-grafted or grafted plants at the same stage of grafting.

### 3.6. Class III Peroxidase

**Figure 7:**
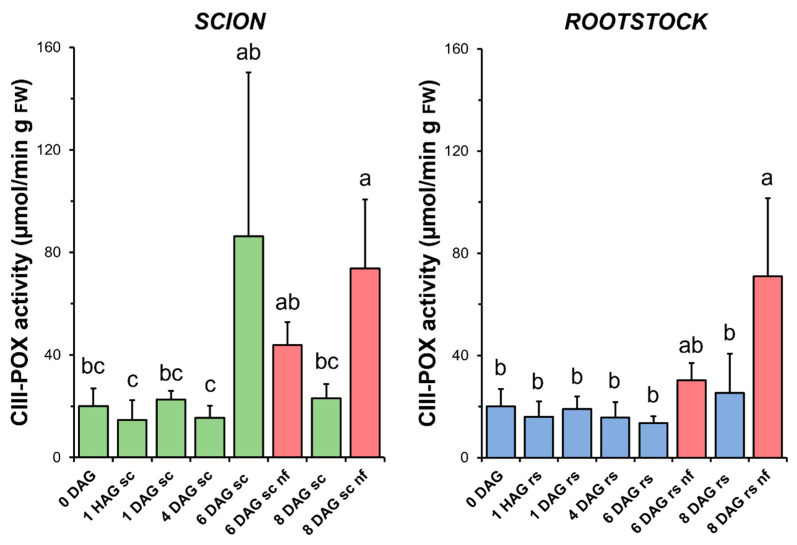
Class III peroxidases (CIII-POX) activity in scion (sc, **green**) and rootstock (rs, **blue**) from 0 to 8 DAG throughout the grafting process. FW, fresh weight. **Red** columns show non-functional grafts (nf). Mean ± SD (n = 5). Different letters indicate significant differences at the *p*-value < 0.05 level after ANOVA analysis of variance followed by Tukey’s test.

CIII-POX activity remained unchanged throughout the graft healing process in both the scions and rootstocks in the functional grafts. In the case of the scions, a non-significant increase in this activity was observed at 6 DAG (Figure 7). Interestingly, a significant increase in CIII-POX activity was measured at 8 DAG in the scion and rootstock tissues from non-functional grafts compared to both the functional grafts at 8 DAG and the non-grafted controls (Figure 7).

### 3.7. Malate Dehydrogenase

**Figure 8:**
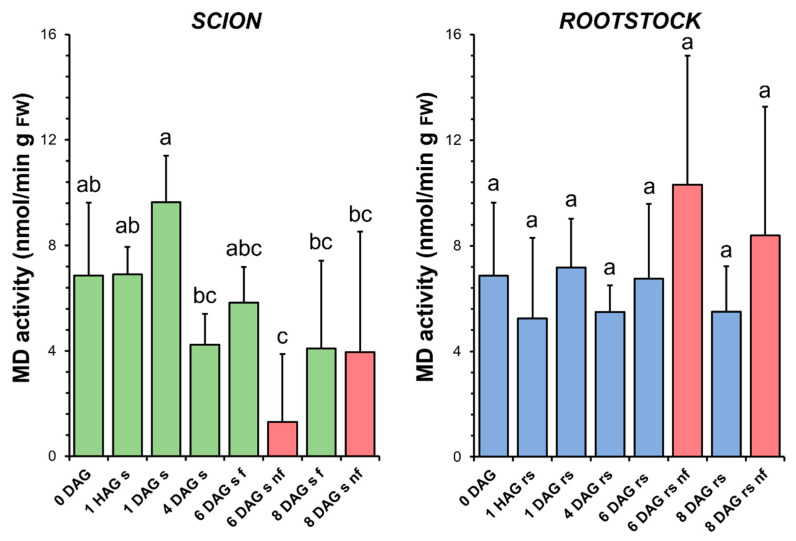
Malate dehydrogenase (MD) activity in scion (sc, **green**) and rootstock (rs, **blue**) from 0 to 8 DAG throughout the grafting process. FW, fresh weight. **Red** columns show non-functional grafts (nf). Mean ± SD (n = 5). Different letters indicate significant differences at the *p*-value < 0.05 level after ANOVA analysis of variance followed by Tukey’s test.

The enzymatic activity of MD underwent some variations throughout the graft healing process in the scions, first an increase at 1 DAG and then a decrease at 4 DAG. It was also found that the specific activity of MD decreased significantly at 6 DAG with respect to the non-grafted plants (Figure 8). In the rootstocks, the level of MD activity did not vary significantly throughout the graft healing process. This activity was only slightly increased in the non-functional rootstocks compared to the non-grafted plants or to the functional grafts at the same stage of grafting (Figure 8).

### 3.8. Ascorbate Peroxidase and Glutathione Reductase

**Figure 9:**
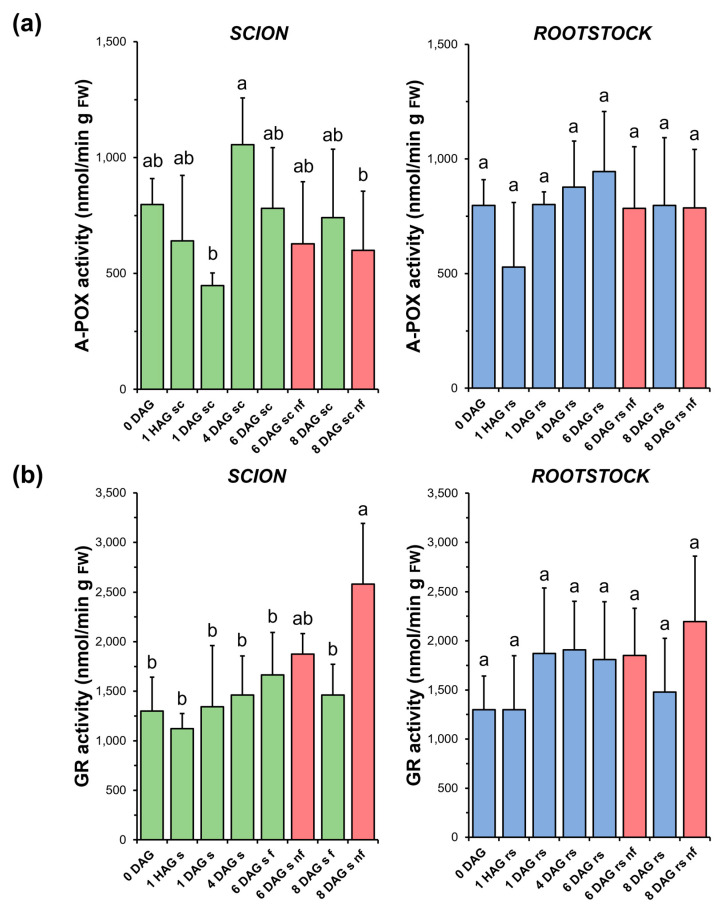
Ascorbate peroxidase (A-POX) (**a**) and glutathione reductase (GR) (**b**) activity in scion (sc, **green**) and rootstock (rs, **blue**) from 0 to 8 DAG throughout the grafting process. FW, fresh weight. **Red** columns show non-functional grafts (nf). Mean ± SD (n = 5). Different letters indicate significant differences at the *p*-value < 0.05 level after ANOVA analysis of variance followed by Tukey’s test.

As shown in Figure 9a, A-POX activity decreased immediately after grafting and then recovered at early DAG in both the scions (4 DAG) and rootstocks (1 DAG). However, due to the variability of the measurements, no significant differences were found in most of the comparisons. In addition, no significant differences were found when comparing the non-functional grafts with the non-grafted or grafted plants at the same stage of grafting.

No significant variations in GR activity (Figure 9b) were observed during the healing process in either functional scion or rootstock samples. However, in the non-functional scions, a significant increase in the activity of this enzyme was observed at 8 DAG compared to the non-grafted or grafted plants at the same stage of grafting. On the other hand, the response of the rootstocks was moderate and similar to the A-POX activity in this type of samples.

### 3.9. Multivariate Analysis: Principal Component Analysis

**Figure 10:**
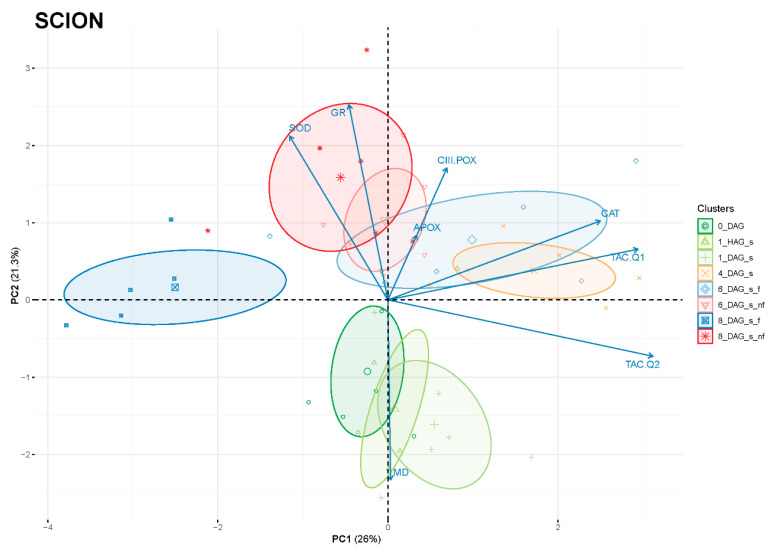
Principal component analysis (PCA) of scion tissues of evaluated variables related to the variation of antioxidant power during the healing process of grafting: total antioxidant capacity (TAC), ascorbate peroxidase (A-POX), glutathione reductase (GR), superoxide dismutase (SOD), catalase (CAT), class III peroxidase (CIII-POX) and malate dehydrogenase (MD) activities. PC1 and PC2 accounted for 47.3% of the variance (26 and 21.3%, respectively). Variables are shown as vectors (arrows). DAG, days after grafting; nf, non-functional; s, scion.

Principal component analysis (PCA) was performed to better interpret the changes in antioxidant activities during the graft healing process. As shown in Figure 10, the clustering of the scions was found to be associated with a timeline of the graft reconnection along PC2 (or even distributed in a counter clockwise pattern starting on the negative side of PC2 with the non-grafted samples and the early times after grafting). Samples corresponding to the initial phase after grafting (0 DAG, 1 HAG and 1 DAG) were located on the negative side of PC2, which showed a correlation with MD activity rather than with SOD, GR, APOX or CIII-POX. Subsequent intermediate times after grafting were located on the positive side of PC2 and on the right side of PC1, with a predominant correlation observed with TAC and CAT. Finally, the last time evaluated of the graft healing process (8 DAG) was positioned away from the intermediate times and in contraposition to antioxidant activities and TAC, suggesting that these are not so necessary when the graft is consolidated.

PCA analysis of rootstock clustering did not show a clear trend, consistent with a high degree of group overlapping (Figure S2).

## 4. Discussion

During graft healing, the wound syndrome together with the onset of water deficit in the scion causes the release of ROS and the activation of antioxidant mechanisms dedicated to controlling oxidative damage [43]. In fact, Cui et al. [12] demonstrated the simultaneous early overexpression of genes related to wound response, oxidative stress and response to water deficit in tomato grafting. If there is an imbalance between the production and scavenging of ROS, the consequences for the success of the graft could be dire. On the one hand, the activation of antioxidant mechanisms is necessary, since the presence of excessive levels of ROS is toxic to the plant, as mentioned above. Nevertheless, ROS contribute to the lignification and/or suberisation of the cutting zone and are used as a signalling/defence mechanism in response to stress and pathogens that attack grafted plants through injured tissues [44,45].

### 4.1. Oxidative Damage Appears to Be Controlled During Grafting in Tissues Close to the Cut Zone

LIPOX is a reliable measure of oxidative stress in grafted plants as it indirectly reflects the formation of hydroxyls by ROS [46]. The results presented here indicate that oxidative damage remained controlled throughout the graft healing process (Figure 3), as no significant differences in LIPOX were found between the non-grafted and grafted plants.

It has previously been shown that highly compatible grafting models have a much greater antioxidant response to ROS than less compatible grafting systems [25,43]. Antioxidant defences help to inactivate the cytotoxic ROS compounds and minimise their ability to diffuse into the intracellular space [24,43]. Therefore, it is possible to suggest that the success of a graft is closely linked to the efficiency of the antioxidant mechanisms and their ability to generate an early response to the wound damage situation [43].

### 4.2. Total Antioxidant Capacity Increases in Tissues Close to the Cut Zone, Likely Controlling Oxidative Damage

In this experimental model, a significant increase in TAC (peaking at 4 DAG) was found in the scion tissues during graft healing (Figure 4). In the case of the rootstock tissues, a non-statistically significant trend towards an increase in TAC was also observed. These results may indicate a link between the induction of a non-enzymatic antioxidant response (Figure 4) and the control of oxidative damage (Figure 3) in successfully autografted plants. These results would be consistent with previous findings indicating a positive relationship between grafting success and an efficient antioxidant response [43].

The eBQC-based-method for TAC measurement assesses the oxidant-buffering capacity of a liquid sample by electrochemical methods. TAC is related to the presence of non-enzymatic antioxidants such as ascorbic acid or glutathione, and, indeed, antioxidant activity is often expressed as ascorbic acid equivalents [47]. One of the main advantages of the eBQC assay for TAC is that it provides information on the contribution of fast (Q1) and slow (Q2) antioxidants [33]. In this regard, our results indicated that slow-acting antioxidants (Q2), which could include compounds such as phenylpropanoids and flavonoids, contributed more to TAC (Figure 4) than fast-acting antioxidants (Q1) such as ascorbic acid [48].

### 4.3. During Graft Healing, Changes in Antioxidant Activities Have Been Observed to Be Associated with Scion Tissues

Although TAC is a rapid and reliable method for monitoring changes in the antioxidant status of a tissue, it does not provide information on the contribution of enzymatic antioxidants. Therefore, an analysis of changes in enzymatic antioxidants during graft healing was performed.

The results presented here highlight the differential contribution of antioxidant activities in the scion and rootstock, emphasising their biochemical diversity during grafting. In functional tomato grafts, it can be concluded that the main variations in antioxidant activities were restricted to the scion tissue (Figure 5, Figure 6, Figure 7, Figure 8 and Figure 9). In addition, principal component analysis revealed a dynamic correlation between antioxidant activities and grafting stages (Figure 10), highlighting their critical role in mitigating oxidative stress.

None of the antioxidant activities evaluated in the rootstocks (SOD, CAT, CIII-POX, MD, A-POX and GR) showed differences with respect to the non-grafted controls during the healing process. A possible explanation for this result is the difference in the level of stress experienced by the rootstock and the scion: the rootstock, which is in contact with the soil and has a source of water and nutrients, would be less stressed than the scion, which is more vulnerable in the phases before the graft is fully healed.

### 4.4. SOD and CAT Activities Increase During Graft Healing in Scion Tissues

The relationship between graft healing and enzymatic antioxidant capacity was particularly well followed in the scion by changes in SOD (Figure 5) and CAT (Figure 6) activities. In both cases, an increase in enzymatic activity was observed over time, with a maximum at 6 DAG. A similar relationship between increases in SOD and CAT and the graft healing process has previously been reported in functional tomato autografts [25].

In this work, oxidative damage was measured by MDA production, a good marker of LIPOX by O_2_^−^ [49,50]. O_2_^−^ is one of the most reactive molecules with the greatest capacity to cause not only lipid degradation but also tissue damage [51]. Furthermore, it should be noted that SODs are the enzymes responsible for the reduction of O_2_^−^ to H_2_O_2_, controlling the accumulation of O_2_^−^ in both the cytoplasm and the cell wall [45,52,53]. Therefore, SOD activity may be responsible for controlling oxidative damage during the graft healing process. O_2_^−^ and H_2_O_2_ are naturally intertwined, as H_2_O_2_ is produced to a greater extent by the SOD-catalysed dismutation of O_2_^−^ [45]. Consequently, an increase in SOD activity is expected to correlate with the accumulation of H_2_O_2_, which is also more stable than another ROS [45]. In this respect, the data show that H_2_O_2_ levels were highly variable immediately after grafting and then decreased to non-grafted levels at late DAG (Figure S1).

H_2_O_2_ is scavenged by peroxisomal/glyoxysomal CAT, among others [45]. The key role of CAT in H_2_O_2_ control has already been demonstrated by using mutants in *Arabidopsis* [54] and in heat-tolerant tomato scions grafted onto a commercial rootstock [55]. As in the case of SOD (Figure 5), the results indicated here point to an increase in CAT activity (Figure 6) up to 6 DAG, also suggesting a role for this enzyme activity in controlling oxidative damage during graft healing, as previously reported for tomato heterografts [25,56]. Similarly, previous reports on tomato grafting have reported an increase in CAT activity

An unexpected finding was observed for catalase activity at 8 DAG, as it was significantly lower in the grafted plants than in the non-grafted plants. In this respect, it is interesting to note that CAT activity is often associated with the accumulation of antioxidant molecules (such as glutathione) during stress situations [57]. The lower catalase activity found in the functional grafted plants compared to the non-grafted plants may therefore indicate an inhibition of activity once oxidative stress has been overcome.

In addition to CAT, other peroxidases such as organellar A-POX and cell wall CIII-POX are involved in controlling H_2_O_2_ levels [45,58]. However, the results obtained here do not allow us to establish a relationship between the variation of CIII-POX (Figure 7) and A-POX (Figure 9a) activities with graft healing in successful grafts.

### 4.5. What About Non-Functional Grafts?

At 6–8 DAG, functional and non-functional grafts could be distinguished morphologically. Taking advantage of this fact, we set out to assess oxidative damage, TAC and enzymatic antioxidant activities in non-functional grafts to evaluate the possible relationship between graft failure and exacerbated oxidative damage.

No differences in the level of oxidative damage (Figure 3) were observed between the scion and rootstock tissues when comparing the non-functional grafts with functional grafts of the same age, although several studies have previously reported an accumulation of H_2_O_2_ causing oxidative damage in incompatible grafts [25,28]. In our work, we also observed few significant differences in TAC (scion at 8 DAG and rootstock at 6 DAG, Figure 4), as well as in SOD (scion at 8 DAG, Figure 5), CAT (scion at 8 DAG, Figure 6) and GR (Figure 9b) activities. These results would suggest that, although there is no causal relationship between graft failure and uncontrolled oxidative stress in our tomato autograft experimental model, changes in antioxidant activities could be a cause or consequence of autograft failure.

CIII-POX is a class of secreted peroxidases involved in cell wall stiffening by oxidatively polymerising lignin, suberin, structural proteins and polysaccharides using H_2_O_2_ as an oxidant [59]. Interestingly, their activity was significantly increased in non-functional grafts (Figure 7). Previous findings allowed us to demonstrate the deposition of defensive cell wall materials, such as suberin and lignin, on the cut surface of non-functional tomato autografts at 20 DAG [60]. It is therefore tempting to hypothesise that the increased CIII-POX activity found here may be related to suberification and lignification during autograft failure.

## 5. Conclusions

The results presented here show that oxidative damage in the stem tissue surrounding the cut surface of autografted tomato plants was not statistically different from that observed in non-grafted control plants during the period up to 8 DAG. This time frame is sufficient for graft healing and consolidation, probably due to the activation of antioxidant mechanisms. An asymmetry in the antioxidant response was reported between the scion and the rootstock, with the scion being the tissue that showed more changes in each of the variables studied. There was a consistent increase in of TAC and CAT and SOD activities as the graft healed. These results suggest that there was an increase in the antioxidant capacity to deal with reactive oxygen species. On the other hand, the non-functional grafts were characterised by an increase in CIII-POX and GR activities. Taken together, these results support the conclusion that the antioxidant enzymatic machinery is altered and enhanced during tomato graft healing and that these changes are also partially different in non-functional grafts. The results presented here suggest that an efficient antioxidant response in scion tissue may be critical for the development of a successful graft.

## Data Availability

The original contributions presented in this study are included in the article and Supplementary Materials. Further inquiries can be directed to the corresponding author.

## Supplementary Materials

The following supporting information can be downloaded at: https://www.mdpi.com/article/10.3390/antiox14020234/s1, Figure S1. Hydrogen peroxide (H_2_O_2_) concentration in scion and rootstock tissues, including non-functional grafts (nf), expressed as percentage of non-grafted plants at 0 days after grafting. Times after grafting evaluated were: 0 days after grafting, 1 h after grafting, 1, 4, 6 and 8 days after grafting. Figure S2. Principal component analysis (PCA) of rootstock tissues of evaluated variables related to the antioxidant power variation during the healing grafting process: total antioxidant capacity (TAC), ascorbate peroxidase (A-POX), glutathione reductase (GR), superoxide dismutase (SOD), catalase (CAT), class III peroxidase (CIII-POX) and malate dehydrogenase (MD) activities. PC1 and the PC2 represented 43.9 % of the variance (23.7 and 20.2 % respectively). Variables are represented as vectors (arrows). DAG, days after grafting; nf, non-functional; rs, rootstock.

## Abbreviations

A-POX	ascorbate peroxidase
CIII-POX	class III peroxidase
CAT	catalase
DAG	days after grafting
GR	glutathione reductase
HAG	hours after grafting
LIPOX	lipid peroxidation
MD	malate dehydrogenase
MDA	malondialdehyde
PCA	principal component analysis
POX	peroxidase
ROS	reactive oxygen species
SOD	superoxide dismutase
TAC	total antioxidant capacity

## References

[1] Feng M., Augstein F., Kareem A., Melnyk C.W. (2024). Plant grafting: Molecular mechanisms and applications. Mol. Plant.

[2] Kyriacou M.C., Rouphael Y., Colla G., Zrenner R., Schwarz D. (2017). Vegetable grafting: The implications of a growing agronomic imperative for vegetable fruit quality and nutritive value. Front. Plant Sci..

[3] Argento S., Treccarichi S., Melilli M.G., Branca F. (2023). Grafting compatibility and environmental conditions on soilless eggplant (*Solanum melongena*) grown in the Mediterranean greenhouse. Horticulturae.

[4] Bento da Silva E.P.P., Mendonça S.R., de Moraes M.G. (2023). Trends and gaps in tomato grafting literature: A systematic approach. Span. J. Agric. Res..

[5] Singh H., Kumar P., Kumar A., Kyriacou M.C., Colla G., Rouphael Y. (2020). Grafting tomato as a tool to improve salt tolerance. Agronomy.

[6] Zeist A.R., Henschel J.M., Silva Júnior A.D., Oliveira G.J.A., Neto J.G., Beauboeuf C.B., Parthasarathi T., de Resendee J.T.V. (2023). Responses of rootstocks variability to tolerate salinity in tomato. S. Afr. J. Bot..

[7] Zhang Z., Liu Y., Cao B., Chen Z., Xu K. (2020). The effectiveness of grafting to improve drought tolerance in tomato. Plant Growth Regul..

[8] Khapte P.S., Kumar P., Wakchaure G.C., Jangid K.K., Colla G., Cardarelli M., Rane J. (2022). Application of phenomics to elucidate the influence of rootstocks on drought response of tomato. Agronomy.

[9] Nakano K. (2021). Mechanisms of resistance to *Ralstonia solanacearum* in tomato rootstocks and integrated management of bacterial wilt using high grafting. J. Gen. Plant Pathol..

[10] Saman P., Kawicha P., Sangdee A., Wongpakdee S., Rattanapolsan L., Ponpang-Nga P., Suwor P., Thanyasiriwat T. (2022). Grafting compatibility, scion growth, and fusarium wilt disease incidence of intraspecific grafted tomato. J. Hortic. Res..

[11] Nanda A.K., Melnyk C.W. (2018). The role of plant hormones during grafting. J. Plant Res..

[12] Cui Q., Xie L., Dong C., Gao L., Shang Q. (2021). Stage-specific events in tomato graft formation and the regulatory effects of auxin and cytokinin. Plant Sci..

[13] Frey C., Manga-Robles A., Acebes J.L., Encina A. (2022). The graft framework: Quantitative changes in cell wall matrix polysaccharides throughout the tomato graft union formation. Carbohydr. Polym..

[14] Loupit G., Brocard L., Ollat N., Cookson S.J. (2023). Grafting in plants: Recent discoveries and new applications. J. Exp. Bot..

[15] Duan Y., Zhang F., Meng X., Shang Q. (2024). Spatio-temporal dynamics of phytohormones in the tomato graft healing process. Hortic. Plant J..

[16] Frey C., Saez-Aguayo S., Encina A., Acebes J.L. (2024). Deepening the role of pectin in the tissue assembly process during tomato grafting. Plants.

[17] Savatin D.V., Gramegna G., Modesti V., Cervone F. (2014). Wounding in the plant tissue: The defence of a dangerous passage. Front. Plant Sci..

[18] Vega-Muñoz I., Duran-Flores D., Fernández-Fernández Á.D., Heyman J., Ritter A., Stael S. (2020). Breaking bad news: Dynamic molecular mechanisms of wound response in plants. Front. Plant Sci..

[19] Chen G., Lips S.H., Sagi M. (2002). Biomass production, transpiration rate and endogenous abscisic acid levels in grafts of flacca and wild-type tomato (*Lycopersicon esculentum*). Funct. Plant Biol..

[20] Correia M.J., Osório M.L., Osório J., Barrote I., Martins M., David M.M. (2006). Influence of transient shade periods on the effects of drought on photosynthesis, carbohydrate accumulation and lipid peroxidation in sunflower leaves. Environ. Exp. Bot..

[21] Frey C., Hernández-Barriuso A., Encina A., Acebes J.L. (2023). Non-invasive monitoring of tomato graft dynamics using thermography and fluorescence quantum yields measurements. Physiol. Plant..

[22] Aroca R., Ruiz-Lozano J.M., Zamarreño A.M., Paz J.A., García-Mina J.M., Pozo M.J., López-Ráez J.A. (2013). Arbuscular mycorrhizal symbiosis influences strigolactone production under salinity and alleviates salt stress in lettuce plants. J. Plant Physiol..

[23] Havaux M. (1993). Rapid photosynthetic adaptation to heat stress triggered in potato leaves by moderately elevated temperatures. Plant. Cell Environ..

[24] Mittler R. (2017). ROS are good. Trends Plant Sci..

[25] Babar M., Afzal N., Siddiqui K., Azhar A., Galani S. (2023). Exploring graft incompatibility markers: Intraspecific and interspecific grafts of tomato (*Solanum lycopersicum* L.). Sci. Hort..

[26] Miller G., Shulaev V., Mittler R. (2008). Reactive oxygen signaling and abiotic stress. Physiol. Plant..

[27] Baxter A., Mittler R., Suzuki N. (2014). ROS as key players in plant stress signalling. J. Exp. Bot..

[28] Aloni B., Karni L., Deventurero G., Levin Z., Cohen R., Katzir N., Lotan-Pompan M., Edelstein M., Aktas H., Turhan E. (2008). Physiological and biochemical changes at the rootstock–scion interface in graft combinations between *Cucurbita* rootstocks and a melon scion. J. Hortic. Sci. Biotechnol..

[29] Noctor G., Foyer C.H. (1998). Ascorbate and glutathione: Keeping active oxygen under control. Annu. Rev. Plant Physiol. Mol. Biol..

[30] Foyer C.H., Kunert K. (2024). The ascorbate–glutathione cycle coming of age. J. Exp. Bot..

[31] Du Z., Bramlage W.J. (1992). Modified thiobarbituric acid assay for measuring lipid oxidation in sugar-rich plant tissue extracts. J. Agric. Food Chem..

[32] Cheeseman J.M. (2006). Hydrogen peroxide concentrations in leaves under natural conditions. J. Exp. Bot..

[33] Aranaz M., Costas-Rodríguez M., Lobo L., González-Iglesias H., Pereiro R., Vanhaecke F. (2022). Homeostatic alterations related to total antioxidant capacity, elemental concentrations and isotopic compositions in aqueous humor of glaucoma patients. Anal. Bioanal. Chem..

[34] Droillard M.J., Paulin A., Massot J.C. (1987). Free radical production, catalase and superoxide dismutase activities and membrane integrity during senescence of petals of cut carnations (*Dianthus caryophyllus*). Physiol. Plant..

[35] Ádám A., Bestwick C., Barna B., Mansfield J. (1995). Enzymes regulating the accumulation of active oxygen species during the hypersensitive reaction of bean to *Pseudomonas syringae* pv. phaeolicola. Planta.

[36] Corpas J.F., Barroso B.F., Sandalio M.L., Distefano S., Palma M.J., Lupiáñez J.A., del Río A.L. (1998). A dehydrogenase-mediated recycling system of NADPH in plant peroxisomes. Biochem. J..

[37] Hossain M.A., Asada K. (1984). Inactivation of ascorbate peroxidase in spinach chloroplasts on dark addition of hydrogen peroxide: Its protection by ascorbate. Plant Cell Physiol..

[38] Edwards E.A., Rawsthorne S., Mullineaux P.M. (1990). Subcellular distribution of multiple forms of glutathione reductase in leaves of pea (*Pisum sativum* L.). Planta.

[39] Kassambara A., Mundt F. *Factoextra: Extract and Visualize the Results of Multivariate Data Analyses*; R Package Version 1; 2020; p. 7. https://cran.r-project.org/web/packages/factoextra/index.html.

[40] Lê S., Josse J., Husson F. (2008). FactorMineR: An R package for multivariate analysis. J. Stat. Softw..

[41] Warnes G., Bolker B., Bonebakker L., Gentleman R., Liaw W.H.A., Lumley T., Magnusson A., Moeller S., Schwartz M., Venables B. *Gplots: Various R Programming Tools for Plotting Data*; R package version 3; 2020; p. 1. https://cran.r-project.org/web/packages/gplots/gplots.pdf.

[42] Frey C., Martínez-Romera N., Encina A., Acebes J.L. (2023). Immunohistochemical dynamics of cell wall matrix polymers during tomato autograft healing. Plant Mol. Biol..

[43] Irisarri P., Binczycki P., Errea P., Juel H., Pina A. (2015). Oxidative stress associated with rootstock–scion interactions in pear/quince combinations during early stages of graft development. J. Plant Physiol..

[44] Minibayeva F., Beckett R.P., Kranner I. (2015). Roles of apoplastic peroxidases in plant response to wounding. Phytochemistry.

[45] Smirnoff N., Arnaud D. (2019). Hydrogen peroxide metabolism and functions in plants. New Phytol..

[46] Pina A., Cookson S.J., Calatayud A., Trinchera A., Errea P., Colla G., Pérez-Alfocea F., Schwarz D. (2017). Physiological and molecular mechanisms underlying graft compatibility. Vegetable Grafting: Principles and Practices.

[47] Pisoschi A.M., Negulescu G.P. (2011). Methods for total antioxidant activity determination: A review. Biochem. Anal. Biochem..

[48] Schauss A.G., Wu X., Prior R.L., Ou B., Huang D., Owens J., Agarwal A., Jensen G.S., Hart A.N., Shanbrom E. (2006). Antioxidant capacity and other bioactivities of the freeze-dried amazonian palm berry, *Euterpe oleraceae* Mart. (Acai). J. Agric. Food Chem..

[49] Del Río D., Stewart A.J., Pellegrini N. (2005). A review of recent studies on malondialdehyde as toxic molecule and biological marker of oxidative stress. Nutr. Metab. Cardiovasc. Dis..

[50] Tsikas D. (2017). Assessment of lipid peroxidation by measuring malondialdehyde (MDA) and relatives in biological samples: Analytical and biological challenges. Anal. Biochem..

[51] Del Río L.A. (2015). ROS and RNS in plant physiology: An overview. J. Exp. Bot..

[52] Apel K., Hirt H. (2004). Reactive oxygen species: Metabolism, oxidative stress, and signal transduction. Annu. Rev. Plant Biol..

[53] Gill S.S., Anjum N.A., Gill R., Yadav S., Hasanuzzaman M., Fujita M., Mishra P., Sabat S.C., Tuteja N. (2015). Superoxide dismutase—Mentor of abiotic stress tolerance in crop plants. Environ. Sci. Pollut. Res..

[54] Queval G., Issakidis-Bourguet E., Hoeberichts F.A., Vandorpe M., Gakiere B., Vanacker H., Miginiac-Maslow M., Van Breusegem F., Noctor G. (2007). Conditional oxidative stress responses in the Arabidopsis photorespiratory mutant *cat2* demonstrate that redox state is a key modulator of daylength dependent gene expression, and define photoperiod as a crucial factor in the regulation of H_2_O_2_-induced cell death. Plant J..

[55] Lee C., Harvey J.T., Qin K., Leskovar D. (2023). Physio-biochemical responses of grafted tomatoes differing in thermotolerance to heat stress and recovery. Sci. Hortic..

[56] Fernández-García N., Carvajal M., Olmos E. (2004). Graft union formation in tomato plants: Peroxidase and catalase involvement. Ann. Bot..

[57] Noctor G., Mhamdi A., Chaouch S., Han Y., Neukermans J., Marquez-Garcia B., Queval G., Foyer C.H. (2012). Glutathione in plants: An integrated overview. Plant Cell Environ..

[58] Wang Y., Wang Q., Zhao Y., Han G., Zhu S. (2015). Systematic analysis of maize class III peroxidase gene family reveals a conserved subfamily involved in abiotic stress response. Gene.

[59] Francoz E., Ranocha P., Nguyen-Kim H., Jamet E., Burlat V., Dunand C. (2015). Roles of cell wall peroxidases in plant development. Phytochemistry.

[60] Frey C., Álvarez R., Encina A., Acebes J.L. (2021). Tomato graft union failure is associated with alterations in tissue development and onset of cell wall defense responses. Agronomy.

