# Supplementary material for: Deciphering Antioxidant Responses in Tomato Autografts
Source: Antioxidants (Basel). 2025 Feb 18;14(2):234. doi: 10.3390/antiox14020234 (PMC11852250; doi:10.3390/antiox14020234)
Supplement: Supplementary file 1 [file antioxidants-14-00234-s001.zip › antioxidants-3422354-supplementary.pdf]

Deciphering antioxidant responses in tomato autografts

Carlos Frey[1,2], Andrés Hernández-Barriuso[1], José Luis Acebes[1,3] and Antonio Encina[1,2*]

[1]Área de Fisiología Vegetal, Facultad de Ciencias Biológicas y Ambientales, Universidad de León, León, España.

[2]Instituto de Biología Molecular, Genómica y Proteómica de la Universidad de León, León, España.

[3]Instituto de la Viña y el Vino de la Universidad de León, León, España.

*Correspondence: AE (a.encina@unileon.es; 0000-0002-1559-1136). Campus de Vegazana, 24007, León (Spain).

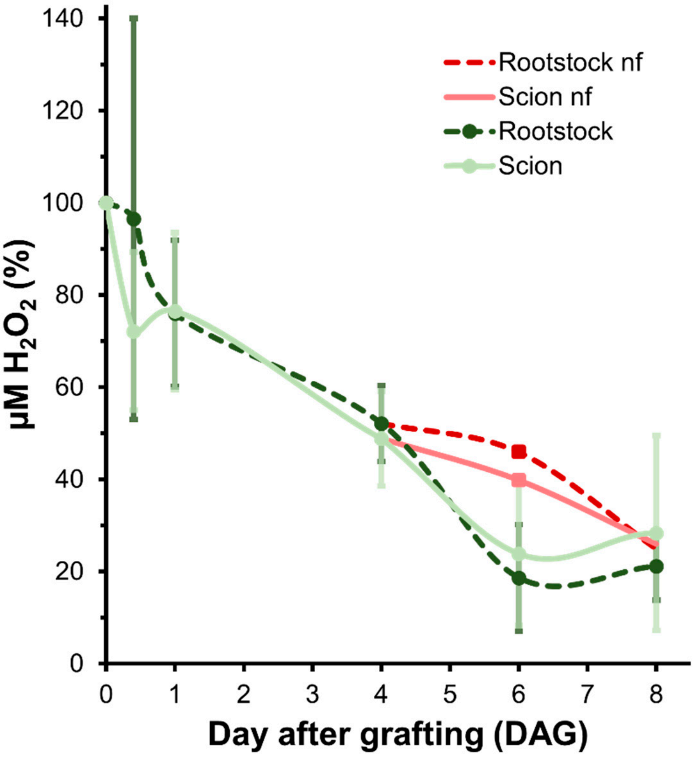

Figure S1. Hydrogen peroxide (H_2O_2) concentration in scion and rootstock tissues, including non-functional grafts (nf), expressed as percentage of non-grafted plants at 0 days after grafting. Times after grafting evaluated were: 0 days after grafting, 1 hour after grafting, 1, 4, 6 and 8 days after grafting.

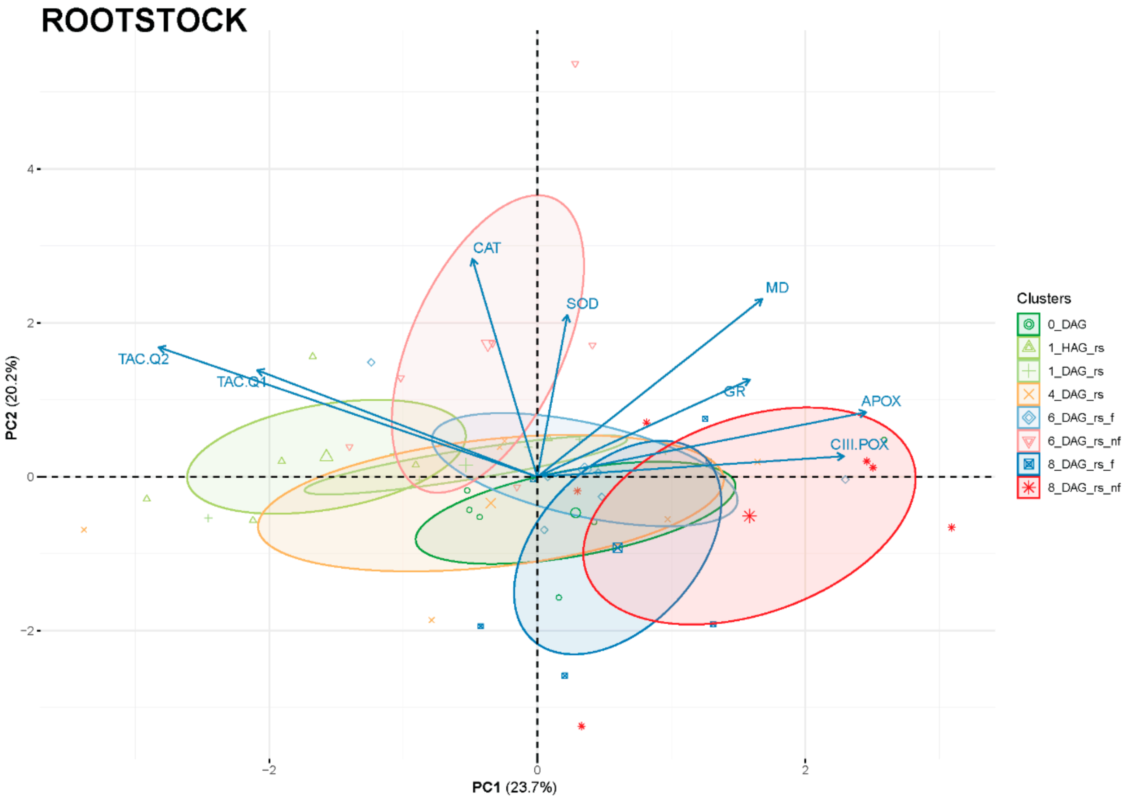

Figure S2. Principal component analysis (PCA) of rootstock tissues of evaluated variables related to the antioxidant power variation during the healing grafting process: total antioxidant capacity (TAC), ascorbate peroxidase (A-POX), glutathione reductase (GR), superoxide dismutase (SOD), catalase (CAT), class III peroxidase (CIII-POX) and malate dehydrogenase (MD) activities. PC1 and the PC2 represented 43.9 % of the variance (23.7 and 20.2 % respectively). Variables are represented as vectors (arrows). DAG, days after grafting; nf, non-functional; rs, rootstock.
